# Methods for Endoscopic Removal of Over-the-Scope Clip: A Systematic Review

**DOI:** 10.1155/2020/5716981

**Published:** 2020-08-23

**Authors:** Ying Hua Ou, Wei Fang Kong, Li Fu Li, Pei Sheng Chen, San Hua Deng, Feng Jian He, Qian Qian Peng, Hui Yue

**Affiliations:** Department of Gastroenterology and Hepatology, The Third Affiliated Hospital of Southern Medical University, Guangzhou, 510630 Guangdong Province, China

## Abstract

**Aims:**

The over-the-scope clip (OTSC) has recently emerged as a new endoscopic device for treating gastrointestinal bleeding, perforations, fistulas, and leaks. A modified OTSC device (full-thickness resection device, FTRD) has been widely used for endoscopic full-thickness resection. However, there is less experience regarding the indications and methods for OTSC removal. We aimed to summarize the existing methods and indications for OTSC removal.

**Methods:**

We searched PubMed, Cochrane Library, and ClinicalTrials.gov to identify relevant publications on OTSC removal. The details of OTSC removal, including the methods, indications, success rates, adverse events, and failure causes, were extracted and summarized. A meta-analysis of pooled success rates was conducted using STATA 15.0.

**Results:**

Eighteen articles were included. The reported methods for OTSC removal included (1) grasping forceps, (2) the Nd : YAG laser, (3) argon plasma coagulation, (4) the remOVE system, (5) endoscopic mucosal resection/endoscopic submucosal dissection, and (6) ice-cold saline solution. Indications for OTSC removal were (1) poor healing, (2) OTSC misplacement, (3) repeat biopsy/therapy or further treatment, (4) adverse events after OTSC implantation, (5) removal after recovery, and (6) patient wishes. The pooled success rate of OTSC removal was 89% in patients treated with the remOVE system. Minor bleeding, superficial thermal damage, and superficial mucosal tears were common adverse events. Mucosal overgrowth was the main cause of OTSC removal failure.

**Conclusions:**

The remOVE system is the best investigated method, with sufficient efficacy and safety for OTSC removal. This is the first systematic review of OTSC removal and provides significant guidance for clinical practice.

## 1. Introduction

The over-the-scope clip (OTSC, Ovesco Endoscopy AG, Tuebingen, Germany) is an innovative endoscopic treatment device that presents several advantages, such as having a powerful clamping ability and a broader range for the closure of gastrointestinal bleeding sites, leaks, and fistulas [1]. The OTSC has also been applied to close wounds after endoscopic full-thickness resection and to anchor esophageal self-expandable metallic stents [2]. Recently, a new over-the-scope system, the full-thickness resection device (FTRD, Ovesco Endoscopy AG, Tuebingen, Germany), which consists of a modified OTSC, a polypectomy snare, and grasping forceps, has been widely used for endoscopic full-thickness resection [3].

Over the past decade, the safety and efficacy of the OTSC in endoscopic treatments have been proven by many clinical trials [2, 4]. However, those trials were conducted with either a small sample size or a short follow-up period. As a result, the safety of long-term OTSC retention inside the human body has not been well illuminated. Unlike normal titanium clips, it is more difficult for the OTSC to spontaneously detach from the mucosa. In some rare cases, OTSC removal might be desirable. These cases included OTSC misplacement or adverse events, such as ulcers and stenosis of the digestive tract [5]. Some patients have even suffered psychosomatic abdominal pain due to awareness of the clips [6]. However, there are neither guidelines nor a consensus concerning the indications or methods for OTSC removal.

Hence, the purpose of this systematic review was to comprehensively summarize the indications for OTSC removal and to assess the experience gained with OTSC removal in terms of the success rates, failure causes, and adverse events of each method.

## 2. Methods

We conducted a comprehensive search of the PubMed, Cochrane Library, and ClinicalTrials.gov databases with the following key words: “over-the-scope clip,” “OTSC,” “Full-Thickness Resection Device,” “FTRD,” “remove,” and “follow up.” The last search was conducted on 20 January 2020. We also reviewed the references of the identified articles to acquire further studies that were not published in the databases mentioned above. The inclusion criteria were as follows: (1) articles published in English; (2) human subjects ≥18 years old; (3) articles published as clinical trials, case reports, or conference abstracts; (4) OTSCs used in the gastrointestinal tract; and (5) available details of OTSC removal needed for analysis. The exclusion criteria were as follows: (1) animal models as subjects; (2) subjects ≤18 years old; (3) unavailable full text of the original article; (4) articles other than a clinical trial, case report, or conference abstract; (5) insufficient information on OTSC removal; and (6) nonendoscopic OTSC removal, such as the removal of an anally placed OTSC by an external clip cutter [7]. The data were extracted by two independent investigators. Disagreements were discussed and resolved with a third investigator.

As the studies included in the meta-analysis were nonrandomized studies, the methodological index for nonrandomized studies (MINORS) was applied for quality assessment (Table 1) [11]. The statistical analyses were performed using STATA 15.1 and Office 365. The chi-squared and I-squared tests were used to calculate the heterogeneity among the studies. The pooled rate and 95% confidence interval were calculated by a fixed model (*I*^2^ < 40%) or a random model (*I*^2^ > 40%). The results are presented as forest plots. A *P* value ≤0.05 was considered statistically significant in this study. *I*^2^ > 40% was considered to indicate high heterogeneity among the included studies.

## 3. Results

### 3.1. Eligible Studies

**Figure 1:**
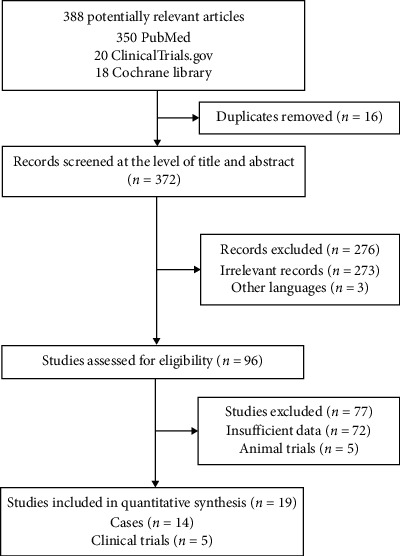
Flowchart for study selection.

**Figure 2:**
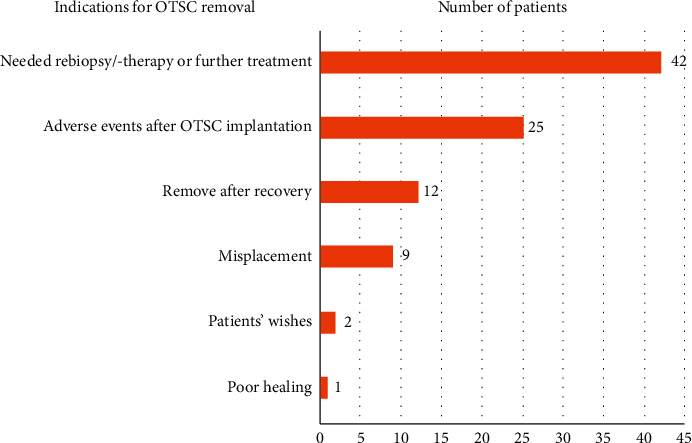
Distribution of indications for OTSC removal across the 17 studies (*n* = 91). Caputo et al. [10] and Mumtaz et al. [17] were not included because indications of OTSC removals were unavailable in the article.

The abstracts of 396 retrieved studies are screened, as shown in Figure 1. After exclusion based on the above criteria, 19 eligible studies were ultimately selected, of which 5 were clinical studies, and 10 were case reports [5, 6, 8–10, 12–21]. One case series [22] and 3 clinical trials [23–25] concerning clinical applications of the OTSC or FTRD were also included because they covered quite a number of clip removal cases during follow-up. We handled these 4 studies as case reports because fewer than 3 removal cases were mentioned in each study. The characteristics of the eligible studies are shown in Tables 2 and 3. The 14 included case reports were published between 2010 and 2018. Altogether, 23 patients aged 21–77 years old were reported in these 14 case reports. The 5 eligible clinical studies were published between 2014 and 2018 and included 144 patients aged 43–89 years old [5, 6, 8–10]. There were 6 methods reported in the eligible studies: (1) grasping forceps, (2) the Nd : YAG laser, (3) argon plasma coagulation (APC), (4) the remOVE system (Ovesco Endoscopy AG, Tuebingen, Germany), (5) endoscopic mucosal resection/endoscopic submucosal dissection (EMR/ESD), and (6) ice-cold saline solution. Indications for OTSC removal were reported in 12 of the 14 studies. We summarized the following indications in descending order according to the number of patients: (1) need for repeat biopsy/therapy or further treatment, (2) adverse events after OTSC implantation, (3) removal after recovery, (4) OTSC misplacement, (5) patient wishes, and (6) poor healing (Figure 2). The longest time that an OTSC remained in situ was 469 days. The shortest episode involved removal of the OTSC immediately after misplacement of the device (Tables 2 and 4).

### 3.2. Success Rate of OTSC Removal

**Figure 3:**
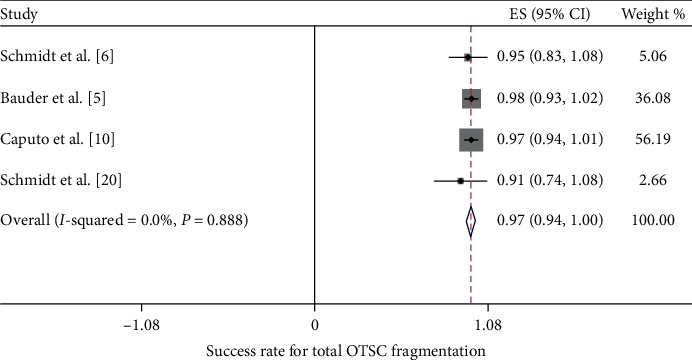
The pooled success rate for OTSC fragmentation by the remOVE system.

**Figure 4:**
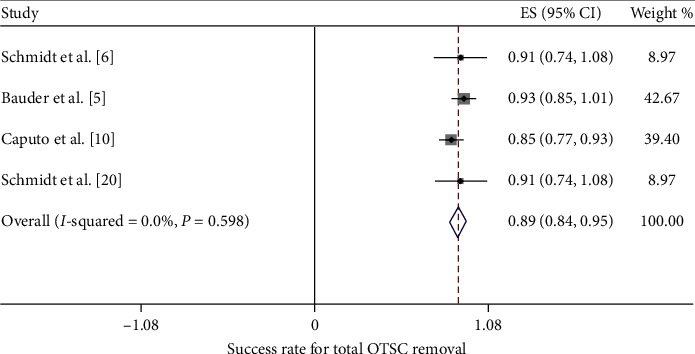
The pooled success rate for total OTSC removal by the remOVE system.

**Figure 5:**
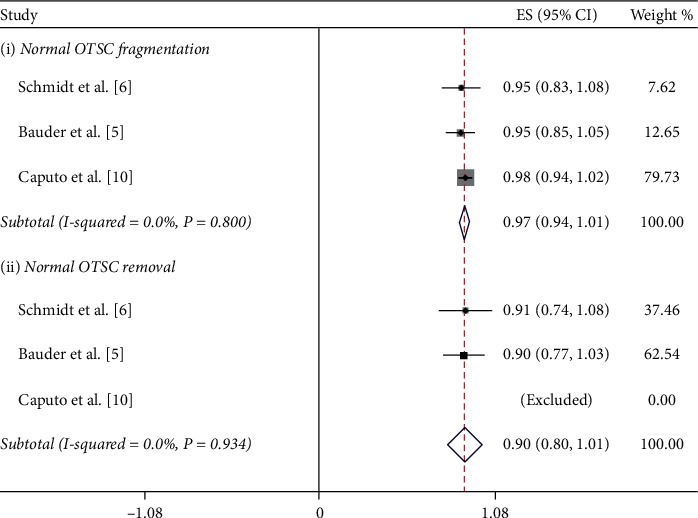
The subgroup analysis of success rate for normal OTSC removal by the remOVE system.

**Figure 6:**
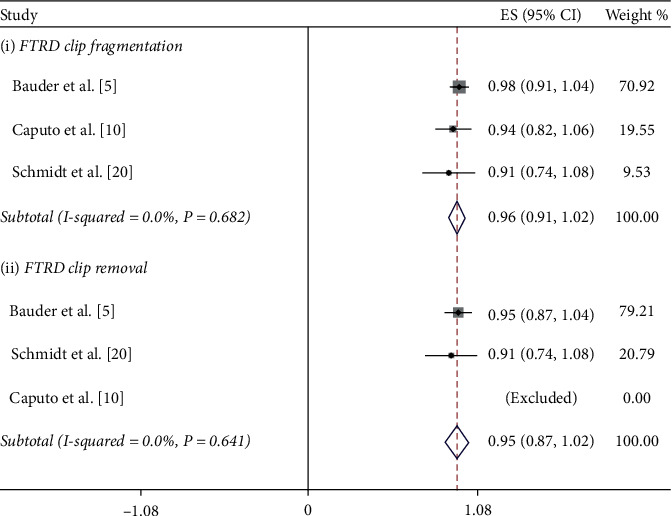
The subgroup analysis of success rate for FTRD clip removal by the remOVE system.

The success rate of OTSC removal in all 14 case reports was 100%. On the other hand, the success rate in the 5 clinical trials ranged from a low of 85% to a high of 100%. The 100% success rate was reported by Mudumb et al., with EMR or ESD as the removal method [8]. The remaining 4 studies, in which the remOVE system was used as the removal method, reported success rates between 85% and 93% [5, 6, 10]. Furthermore, we performed a meta-analysis of these 4 studies. The procedure for the removal of an OTSC by the remOVE system involves two main steps. The first step, called fragmentation, is to cut the OTSC into two pieces at the bow on both sides using a DC cutter. The second step, known as retrieval, is to grasp and remove the two fragments through an endoscopic grasper. We calculated the pooled success rate of both steps. The pooled success rate for overall OTSC fragmentation and retrieval was 97.2% (CI = (0.94, 1.0), *P* < 0.001, *I*^2^ = 0.0%, *P*_het_=0.888) and 89.5% (CI = (0.84, 0.95), *P* < 0.001, *I*^2^ = 0.0%, *P*_het_=0.598), respectively (Figures 3 and 4). Specifically, the FTRD is designed differently than the normal OTSC in terms of size and teeth, which might affect the success rate. As a result, we conducted a subgroup analysis according to the type of clip. The pooled success rate for normal OTSC fragmentation and retrieval was 97.5% (CI = (0.94, 1.01), *P* < 0.001, *I*^2^ = 0.0%, *P*_het_=0.80) and 90.3% (CI = (0.80, 1.01), *P* < 0.001*I*^2^ = 0.0%, *P*_het_=0.934), respectively (Figure 5). The pooled success rate for FTRD fragmentation and retrieval was 96.3% (CI = (0.91, 1.02), *P* < 0.001, *I*^2^ = 0.0%, *P*_het_=0.682) and 94.5% (CI = (0.87, 1.02), *P* < 0.001, *I*^2^ = 0.0%, *P*_het_=0.641), respectively (Figure 6). When calculating the removal rate in the normal OTSC and FTRD groups, the Caputo study [10] was excluded because the success rates of the two clip types had not been discussed separately.

### 3.3. Adverse Events after OTSC Removal

We intended to discriminate between minor and major adverse events according to the need for further endoscopic treatment. Events that did not need special treatment, such as minor bleeding, superficial mucosal tears, and superficial mucosal thermal damage, were defined as minor adverse events. These minor adverse events usually have no consequences concerning patient management or outcome. In contrast, major adverse events were considered as those that needed further intervention, such as major bleeding, delayed bleeding, and perforation.

**Figure 7:**
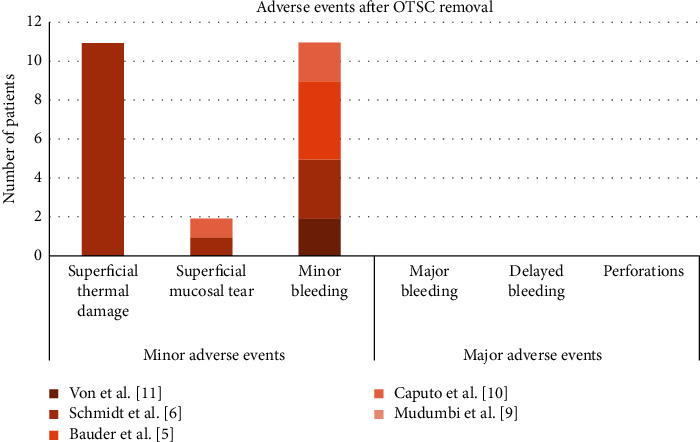
Distribution of adverse events after removal of OTSC across 5 studies (*n* = 24).

**Figure 8:**
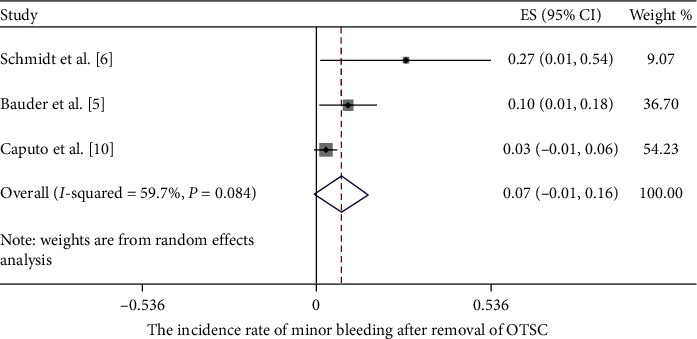
The pooled incidence rate of minor bleeding after OTSC removal.

Adverse events after OTSC removal were reported in 5 of the included studies (Figure 7) [5, 6, 8, 10, 12]. There was no record of major adverse events. Regarding minor adverse events, minor bleeding was reported in 3 clinical trials [5, 6, 10], with a pooled incidence rate of 7% (CI = (−0.01, 0.16), *P*=0.09, *I*^2^ = 59.7%, *P*_het_=0.084) (Figure 8). Superficial thermal damage was only reported by Schmidt et al. [6] who used the remOVE system as the removal method, with an incidence rate of 100%. Superficial mucosal tears were reported by Caputo et al. [10] and Schmidt et al. [6], with an incidence rate of 1.4% and 9.1%, respectively. However, Mudumbi et al. [8] declared no adverse events after OTSC removal through EMR or ESD. All adverse events were repaired through endoscopic treatment. No long-term adverse events were recorded.

### 3.4. Failure of OTSC Removal

In summary, 16 cases of procedural failure were recorded in 4 remOVE system studies [5, 6, 9, 10]. The cases in the remaining 15 studies were all successful. In three of the 15 cases, failure occurred at the fragmentation step because the OTSC had been deeply implanted into the mucosa and could not be approached from a favorable angle. In the other 12 cases, failure occurred at the retrieval step because the dissected fragments were wrapped by the overgrown tissue. In conclusion, the main cause of removal failure was overgrowth of the gastrointestinal mucosa. The reason for the failure of methods other than the remOVE system was unclear because of the lack of a study population.

## 4. Discussion

### 4.1. Necessity of and Indications for OTSC Removal

The OTSC is made of nitinol, which has favorable biocompatibility [26]. As a result, this device was initially regarded as a permanent implanted material for endoscopic treatment. However, an implanted OTSC might spontaneously detach in the long term. The rate of spontaneous OTSC detachment was 13%, 25%, 50%, and 75% for the gastric fundus, antrum, cardia, and body, respectively, over an average follow-up of 20 months [27]. A systematic review of the OTSC system in the endoscopic closure of iatrogenic gastrointestinal perforations summarized the spontaneous detachment rate as ranging from 0% to 44.4% during 1–12 weeks [4]. Regarding the FTRD, the reported detachment rate mainly ranged from 70% to 80% in the colorectum after 3 months of follow-up [9, 28]. The type of clip might be one of the important factors affecting the spontaneous detachment rate. The FTRD is designed for endoscopic full-thickness resection and is equipped with a larger 14 mm OTSC clip with more teeth. The FTRD clip is usually more difficult to disintegrate with the removal device because its branches are thicker. In addition, according to variations in the amount of tissue grasped within the clip, some superficially implanted OTSCs might more easily detach spontaneously.

Although the OTSC was designed to remain in situ permanently, the safety of long-term OTSC retention is still unknown due to the short follow-up period of the current studies. OTSC removal should be initiated under particular circumstances depending on individual differences. However, there is a need for guidelines or a comprehensive review concerning indications for OTSC removal. To provide guidance for clinical practice, we conducted a comprehensive search for studies on OTSC removal and identified the following indications: (1) need for repeat biopsy/therapy or further treatment, (2) adverse events after OTSC implantation, (3) removal after recovery, (4) OTSC misplacement, (5) patient wishes, and (6) poor healing.

We have also discussed the timing of removal. If removed too soon, the gastrointestinal lesion might heal incompletely. However, if the OTSC remains in situ for too long, the overgrown tissue might impede the removal procedure. Based on the current studies, it is difficult to draw a conclusion on the timing of removal for each type of lesion treated using the OTSC. Recently, the earliest removal time was immediately after implantation in the case of misplacement [12]. Otherwise, the timing of removal ranged from 21 days to 469 days [5, 6, 8, 13, 15, 16, 19, 20]. Bauder et al. found that the mean duration of in situ OTSC retention was not significantly different in the 5 mucosal overgrowth cases of the 42 cases of OTSC implantation (104 days vs. 99 days) [5]. Specifically, for endoscopic full-thickness resection, the FTRD clip should remain in situ for at least 2 months and better 3 months to ensure wound healing [29]. Schmidt et al. suggested that if repeat biopsy/therapy of an adenoma resection site was required, then the FTRD clip should be removed after at least 3 months. However, if the OTSC was implanted to anchor an esophageal self-expandable metallic stent, then the time span should be 6–8 weeks. For other conditions, clip removal was not suggested within 4 weeks [6].

### 4.2. OTSC Removal Methods

According to recent studies, there are 6 main removal methods: (1) grasping forceps, (2) the Nd : YAG laser, (3) APC, (4) the remOVE system, (5) EMR/ESD, and (6) ice-cold saline solution.

#### 4.2.1. Endoscopic Grasping Forceps

Among all the included studies, the use of grasping forceps was reported in only one case report containing two cases [12]. Removal of the OTSC in the two cases occurred because of the incompletely grasped tissue, and the defects were only partially closed. On this occasion, the OTSC could be pulled out easily using endoscopic grasping forceps, leaving a mild superficial tear in the mucosa. Therefore, endoscopic grasping forceps are applied to clips that are placed too superficially or only partially cover the lesion and should be removed immediately after implantation. If the clips remain in situ for a long time are placed at a proper depth, or become wrapped by overgrown mucosa, pulling out an OTSC with endoscopic grasping forceps directly will cause great damage and is not recommended.

#### 4.2.2. Clip Cutting Devices

The Nd : YAG laser, APC, and the remOVE system are three main clip cutting devices that have been described in the present studies [5, 6, 9, 10, 13, 19, 20, 22–25]. The main procedure for OTSC removal using clip cutting devices can be concisely summarized as fragmentation and retrieval. Despite the diversity of the devices, their fragmentation steps are essentially the same, that is, the OTSC is cut into two pieces at the thinnest part, called the bow of the clip, on both sides of the device. Then, the two fragments are separately pulled out using an endoscopic grasper [30]. Among the three devices, the Nd : YAG laser and APC were reported in only one case report, reflecting very low-grade evidence. The remOVE system, a product of Ovesco Endoscopy AG, has been proven to be safe and effective in animal studies and clinical studies [5, 6, 10, 30]. The results of our study show a pooled success rate of 97% and 89% for fragmentation and retrieval, respectively. Mucosal overgrowth was the main cause of failure because the OTSC was buried into the surrounding tissue too deeply to be grasped or approached from a favorable angle. In the subgroup analysis, the success rate of clip fragmentation and removal was 97% and 90% for the normal OTSC and 96% and 95% for the FTRD clip, respectively. Unfortunately, we could not directly compare the removal success rate between the FTRD clip and the normal OTSC based on recent data. Comparative studies focusing on differences between the FTRD clip and the OTSC are needed to determine the difference in the removal success rate. In contrast with other methods, the remOVE system is a well-studied, reliable, safe, and effective method for removing an OTSC.

#### 4.2.3. EMR/ESD

Removing an OTSC through EMR/ESD involves thoroughly excising the OTSC along with the surrounding tissue from the mucosal or submucosal layer, similar to the resection of gastrointestinal masses [31]. In our opinion, EMR/ESD is appropriate for removing OTSCs implanted no deeper than the submucosal layer. As these clips reach the muscular layer, the risks of the removal procedure increase, and adverse events such as major bleeding and perforation are more likely to occur. Additionally, EMR/ESD is the most invasive removal method because it creates a new gastrointestinal defect and may require further OTSC treatment in the case of severe adverse events [32].

#### 4.2.4. Ice-Cold Saline Solution

The OTSC is made of nitinol, a material that becomes malleable at temperatures below 10°C but hardens after the reapplication of heat [26]. Because of its physical peculiarity, cooling the OTSC below 10°C with ice-cold saline solution leads to it becoming soft and deformable, allowing it to be pulled out with a very low force [15]. Although OTSC removal with ice-cold saline solution is easy, the safety and efficacy of this method are uncertain, having been described in only three cases [15, 18, 21]. If the OTSC is incompletely cooled and thus insufficiently softened, removal of the OTSC by force may lead to major bleeding, severe mucosal lacerations, or perforations.

### 4.3. Adverse Events

The main adverse events after OTSC removal were minor adverse events, including minor bleeding, superficial thermal damage, and superficial mucosal tears, reported in studies using the remOVE system and grasping forceps as the removal methods (Figure 7) [5, 6, 10, 12]. The remOVE system consists of two electrodes [30]. The short DC impulse applied during the fragmentation step would likely cause thermal damage. Additionally, the edges of the fragments may sometimes be sharp enough to cause mucosal tears or bleeding, which could be prevented with the correct usage of a secure cap [10]. There were no major adverse events reported in recent studies. However, this finding does not mean that severe adverse events never occur during OTSC removal or that other methods are better than the remOVE system because some researchers might be reluctant to publish their failed results.

### 4.4. Innovations and Limitations

Neither recent guidelines nor a consensus concerning OTSC removal has been published. Over the past decade, researchers and manufacturers have proposed several methods for clip removal. As the first systematic review of this field, we summarized the existing methods for OTSC removal as well as the corresponding indications, success rates, failure causes, and adverse events. The comprehensive analysis and quantitative results of the present study are superior to the content of general reviews. All the above findings have important implications for medical practice and can serve as a reference for clinicians.

However, the included studies were mainly retrospective studies or case reports, which would lead to an increase in selection bias and publication bias and impair the reliability and accuracy of our study. Additionally, we were not able to assess the pooled success rates, safety, or adverse events of methods other than the remOVE system, as the analysis was limited by the type and sample size of the original studies. Clinical trials with large study populations are needed to confirm the safety, efficacy, and long-term adverse events of each method.

### 4.5. Publication Bias

As the number of studies included in the meta-analysis was less than 5, it was inappropriate to assess publication bias quantitatively. However, it is obvious that cases of success are more likely to be published than cases of failure [33]. In particular, in the 14 successful case reports, the success rates were as high as 100%, and very few adverse events were reported. As a result, the success rate might be overestimated. To precisely evaluate the safety and efficacy of each removal method, further clinical trials with large populations are needed.

## 5. Conclusions

Over the past decade, the OTSC has been widely used in clinical practice. Although the OTSC was designed to be available for permanent implantation, there are occasions upon which the OTSC needs to be removed. OTSC removal methods include grasping forceps, the Nd : YAG laser, APC, the remOVE system, EMR/ESD, and ice-cold saline solution. The remOVE system was the most commonly reported method. To date, there is insufficient data for alternative methods. Based on the current status, our study shows that the remOVE system is the best investigated method with sufficient efficacy and safety. The main cause of removal failure was mucosal hyperplasia. Short-term adverse events were minor bleeding, superficial thermal damage, and superficial mucosal tears, and no long-term adverse events have been reported. Our study is the first systematic review of OTSC removal. Multicenter trials of each method with larger populations are needed to provide more reliable guidance for clinical practice.

## Figures and Tables

**Table 1:** Assessment of study quality using methodological index for nonrandomized studies (MINORS).

	Mudumbi et al.[8]	Schmidt et al. [6]	Bauder et al. [5]	Schmidt et al. [9]	Caputo et al. [10]
A clearly stated aim	2	2	2	2	2
Inclusion of consecutive patients	2	2	2	2	2
Prospective collection of data	2	2	2	2	2
Endpoints appropriate to the aim of the study	1	2	2	1	2
Unbiased assessment of the study endpoint	0	0	0	0	0
Follow-up period appropriate to the aim of the study	0	0	0	0	0
Loss to follow-up less than 5%	0	0	1	0	1
Prospective calculation of the study size	0	0	0	0	0
Total score	7	8	9	7	9

The items are scored 0 (not reported), 1 (reported but inadequate), or 2 (reported and adequate). The total ideal score for each noncomparative study is 16.

**Table 2:** Characteristics of the included case reports.

Author	Patients included	Age	Gender (M/F)	Indication		Mean time of OTSC in situ	Success (Y/N)	Adverse events
				For OTSC implantation	For OTSC removal			
(i) *Grasping forceps*								
von Renteln et al. [12]	2	55, 75	M	Esophagopulmonary fistula; jejunocutaneous fistula	Misplacement: partially cover the fistula	Removed immediately after misplacement	Y	2 minor bleeding

(ii) *Nd : YAG-laser*								
Fähndrich et al. [13]	3	61–72	1 F, 2 M	Gastrocutaneous fistula and perforation	1 misplacement: causing a severe esophageal stenosis; 2 removal after recovery	2 months	Y	UA

(iii) *APC*								
Kapadia et al. [20]	1	71	M	Anchored the SEMS for esophagojejunal anastomotic leak	Removal after recovery	2 months	Y	UA

(iv) *The remOVE system*								
Schiffmann et al. [19]	1	21	UA	Gastrocutaneous fistula	Poor healing, needs further OTSC	UA	Y	UA
Meier et al. [24]	2	UA	UA	EFTR	Repeat biopsy after EFTR	3 months	Y	UA
Andrisani et al. [23]	2	UA	UA	EFTR	Repeat therapy after EFTR	3 months	Y	UA
Valli et al. [25]	3	UA	UA	EFTR	R1 resection	4–6 weeks	Y	UA
Mangas-Sanjuan et al. [22]	2	51, 58	1 M, 1 F	Esophageal variceal bleeding	Adverse events: esophageal stenosis	5–8 months	Y	N

(v) *EMR/ESD*								
Mönkemüller et al. [14]	1	77	M	Anastomotic leak after esophagectomy	Misplacement: needs to place a new clip	10 days	Y	UA
Sedarat et al. [16]	1	56	F	Chronic gastrocutaneous fistula	Adverse events: causing pyloric obstruction	UA	Y	UA
Mumtaz et al. [17]	2	UA	UA	UA	UA	UA	Y	UA

(vi) *Ice-cold saline solution*								
Arezzo et al. [15]	1	UA	UA	Rectal chronic fistula	Misplacement	UA	Y	UA
Krishna and Shakhatreh [18]	1	UA	UA	Gastrocutaneous fistula	Misplacement	UA	Y	UA
Rocha et al. [21]	1	51	M	Esophageal fistula	2 OTSC attached to each other causing luminal obstruction	6 weeks	Y	UA

Studies were sorted by removal methods of clips. M, male; F, female; Y, yes; N, no; UA, details were unavailable in the original articles; EMR, endoscopic mucosal resection; ESD, endoscopic submucosal dissection; APC, argon plasma coagulation; SEMS, self-expandable metallic stent; EFTR, endoscopic full-thickness resection.

**Table 3:** Characteristics of the included clinical trials.

Author	Article type	Number of included patients	Age	Gender (M/F)	Indications for OTSC implantation	Indications for OTSC removal
Mudumbi et al. [8]	Observational, retrospective	6	57 (45–72)	8/4	Anchored the esophageal SEMS for fistulas or leaks	6 removal of SEMS after recovery

Schmidt et al. [6]	Retrospective	11	62 (43–73)	7/4	3 perforation; 4 ulcer bleedings; 2 mucosal defects; 1 EFTR; 1 SEMS	6 adverse events after OTSC implantation (2 ulcer, 3 obstruction, and 1 dysphagia); 2 needed repeat biopsy; 1 removal of SEMS after recovery; 2 patients' wishes (all causing psychosomatic abdominal pain)

Bauder et al. [5]	Retrospective	42	65 (35–89)	28/14	UA	22 repeat biopsy/therapy after EFTR 15 adverse events after OTSC implantation (2 ulcer, 9 dysphagia, and 4 abdominal pain); 4 misplaced OTSC; 1 removal of SEMS after recovery

Caputo et al. [10]	Retrospective	74	UA	UA	51 OTSC; 16 FTRD	UA

Schmidt et al. [9]	Retrospective	11	UA	UA	11 FTRD	R1/Rx resection, needs retherapy after EFTR

M, male; F, female; SEMS, self-expandable metallic stent; EFTR, endoscopic full-thickness resection; UA, details were unavailable in the original articles; ^#^FTRD, full-thickness resection device.

**Table 4:** Details of OTSC removal of the included clinical trials.

Author	Mean time of OTSC in situ: days (range)	Mean procedure time: min (range)	Number of included patients	Success		
				Success-cut *n* (%)	Success-retrieve *n* (%)	Total success *n* (%)
(i) *EMR/ESD*						
Mudumbi et al. [8]	180 (21–300)	UA	6	—	—	6 (100%)
(ii) *The remOVE system*						
Schmidt et al. [6]	138 (31–469)	47 (35–75)	11	11 (100%)	10 (90.9%)	10 (90.9%)
Bauder et al. [5]	99 (1–469)	Upper GI tract 47 (20–100); Lower GI tract 58 (40–75)	42	41 (97.6%)	39 (92.9%)	39 (92.9%)
Caputo et al. [10]	UA	UA	74	72 (97.3%)	63 (85.1%)	63 (85.1%)
Schmidt et al. [9]	90	UA	11	10 (90.9%)	10 (90.9%)	10 (90.9%)

Studies were sorted by removal methods of clips. EMR, endoscopic mucosal resection; ESD, endoscopic submucosal dissection; UA, details were unavailable in the original article.

## Data Availability

The data used to support this study are available online and are made available from the corresponding author upon request.
